# Lead-Free Cesium Titanium Bromide Double Perovskite Nanocrystals

**DOI:** 10.3390/nano11061458

**Published:** 2021-05-31

**Authors:** G. Krishnamurthy Grandhi, Anastasia Matuhina, Maning Liu, Shambhavee Annurakshita, Harri Ali-Löytty, Godofredo Bautista, Paola Vivo

**Affiliations:** 1 Hybrid Solar Cells, Faculty of Engineering and Natural Sciences, Tampere University, P.O. Box 541, FI-33014 Tampere, Finland; murthy.grandhi@tuni.fi (G.K.G.); anastasiia.matiukhina@tuni.fi (A.M.); maning.liu@tuni.fi (M.L.); 2 Photonics Laboratory, Physics Unit, Faculty of Engineering and Natural Sciences, Tampere University, FI-33014 Tampere, Finland; shambhavee.annurakshita@tuni.fi (S.A.); godofredo.bautista@tuni.fi (G.B.); 3 Surface Science Group, Photonics Laboratory, Tampere University, P.O. Box 692, FI-33014 Tampere, Finland; harri.ali-loytty@tuni.fi

**Keywords:** lead-free halide perovskites, nanocrystals, titanium (Ti), double perovskites, stability, third-harmonic generation, nonlinear optics

## Abstract

Double perovskites are a promising family of lead-free materials that not only replace lead but also enable new optoelectronic applications beyond photovoltaics. Recently, a titanium (Ti)-based vacancy-ordered double perovskite, Cs_2_TiBr_6_, has been reported as an example of truly sustainable and earth-abundant perovskite with controversial results in terms of photoluminescence and environmental stability. Our work looks at this material from a new perspective, i.e., at the nanoscale. We demonstrate the first colloidal synthesis of Cs_2_TiX_6_ nanocrystals (X = Br, Cl) and observe tunable morphology and size of the nanocrystals according to the set reaction temperature. The Cs_2_TiBr_6_ nanocrystals synthesized at 185 °C show a bandgap of 1.9 eV and are relatively stable up to 8 weeks in suspensions. However, they do not display notable photoluminescence. The centrosymmetric crystal structure of Cs_2_TiBr_6_ suggests that this material could enable third-harmonic generation (THG) responses. Indeed, we provide a clear evidence of THG signals detected by the THG microscopy technique. As only a few THG-active halide perovskite materials are known to date and they are all lead-based, our findings promote future research on Cs_2_TiBr_6_ as well as on other lead-free double perovskites, with stronger focus on currently unexplored nonlinear optical applications.

## 1. Introduction

One of the key obstacles to the practical application and commercialization of metal halide perovskites is the toxicity of their key lead (Pb) constituent [1]. The detrimental effects of Pb on both the environment and human health pose big concerns for its utilization, as highlighted by existing regulations on the use of heavy metals in force in several countries [2]. Hence, there is a constantly growing interest in novel lead-free halide perovskite designs, both as bulk films and nanocrystals (NCs) [3]. However, nearly all the explored alternatives to Pb are based on materials with debatable toxicity, mediocre stability, and/or limited availability, raising disposal and recycling issues [4,5].

Titanium (Ti) is an eco-friendly and earth-abundant element that could potentially be an ideal constituent of sustainable perovskite compositions. Recently, Ju et al. investigated a new family of vacancy-ordered double perovskites based on Ti (IV) with chemical formula A_2_TiX_6_. With a combined theoretical and experimental work, Cs_2_TiBr_6_ perovskite (band gap around 1.8 eV) has proven to be promising for photovoltaic applications [6]. In another report, the same authors showed that Cs_2_TiBr_6_ thin films display efficient photoluminescence (PL), long carrier-diffusion lengths, and energy levels suitable for tandem photovoltaic applications [7]. Moreover, when introduced in solar cells, Cs_2_TiBr_6_ led to a stable power conversion efficiency (PCE) up to 3.3%, which is among the highest PCE values reported for double perovskites. The authors also emphasized the superior intrinsic and environmental (heat, moisture, and light) stability of Cs_2_TiBr_6_, which was synthesized via a vapor deposition method at high temperatures.

These first studies on Ti-based perovskites, highlighting the potential for the development of truly eco-friendly and stable solar cells, have raised a lot of interest in the research community and encouraged other researchers to focus on this family of double perovskites. Very recently, Kong et al. proposed a new solution-processed method at low temperatures for the synthesis of Cs_2_TiBr_6_ [8]. However, almost simultaneously, Euvrard et al. raised some doubts on the actual suitability of this material for photovoltaic applications, given its weak PL and the high instability in ambient conditions [9].

To clarify the dispute on the promise of Cs_2_TiBr_6_ lead-free material for optoelectronics, we aimed at further looking at this material from another perspective, i.e., at the nanoscale. Our motivation to develop Cs_2_TiBr_6_ as NCs was triggered by the known advantages of NCs compared to their bulk counterparts, such as the typical enhanced and tunable optical properties, particularly in terms of higher PL quantum yield, as well as the higher stability [10,11].

In this work, we report the first synthesis of Cs_2_TiBr_6_ NCs and examine the effect of synthesis parameters, such as reaction temperature and capping ligands, on the structural stability and morphology of Cs_2_TiBr_6_ NCs. We also tuned the halide in the NC composition, by synthesizing the chloride-analogue, Cs_2_TiCl_6_, and attempting the synthesis of Cs_2_TiI_6_ NCs, which was, however, only partially successful.

The Cs_2_TiBr_6_ NCs exhibit negligible PL and moderate stability, thus confirming their intrinsic incompatibility with photovoltaic applications. On the other hand, the recent growing interest in exploiting halide perovskites for nonlinear optics [12,13] and the centrosymmetric crystal structure of Cs_2_TiBr_6_ inspired us to investigate the possible third-harmonic generation (THG) response of this material [14]. THG observation and, most importantly, its manipulation is scarcely explored in the context of halide perovskites [15]. Only a few THG-active halide perovskite materials are known to date, and they are all based on Pb [14,16,17,18]. Therefore, there is a big opportunity to develop new or explore existing halide perovskite compositions with exceptional THG responses. Our early findings show the observation of THG signals in Cs_2_TiBr_6_ by THG microscopy, in turn demonstrating the potential of lead-free perovskite NCs, and particularly Ti-based ones, as THG-active compounds. This work provides a promising alternative to the already explored photovoltaic application for Cs_2_TiBr_6_ and suggests a stronger focus in this direction for this perovskite material as well as for other lead-free double perovskites.

## 2. Materials and Methods

### 2.1. Materials

Cs_2_CO_3_ (99.9%), octadecene (ODE, 90%), oleic acid (OA, 90%), oleylamine (OlAm, technical grade, 70%), titanium di-isopropoxide bis(acetylacetonate) (TDBA) 75 wt.% in isopropanol, Chlorotrimethylsilane (TMS-Cl, ≥99%), bromotrimethylsilane (TMS-Br, purum, ≥97.0% (AT)), and iodotrimethylsilane (TMS-I, 97.0%) were purchased from Sigma-Aldrich (St. Louis, MO, USA). All chemicals were used without further purification.

### 2.2. Synthesis of Cs_2_TiBr_6_ and Cs_2_TiCl_6_ NCs

For the colloidal synthesis of Cs_2_TiBr_6_ NCs, di-isopropoxide bis(acetylacetonate) (TDBA; 50 µL, 0.10 mmol), Cs_2_CO_3_ (35 mg, 0.10 mmol), ODE (8.0 mL), OlAm (0.60 mL), and OA (1.0 mL) were loaded into a 25 mL three-neck round-bottom flask and dried by refluxing under vacuum for 2 h while magnetically stirring at 350 rpm on a magnetic stirrer. To the clear precursor solution, TMS-Br (0.6 mL) was swiftly injected at different temperatures (90, 135, 185, and 245 °C) under Ar flow, which resulted in a color change of precursor solution to bright red, instantaneously. The reaction flask was kept at the injection temperature for 10–15 s before an ice-water bath was set under the three-neck flask to quench the further growth of NCs. The as-obtained colloidal suspension was centrifuged at 4500 rpm for 5 min. The supernatant was discarded, and the precipitate containing NCs was dispersed in 3 mL of hexane. The colloidal solution of Cs_2_TiBr_6_ NCs was stored inside the glovebox until further use. Cs_2_TiCl_6_ NCs were prepared by following the above procedure except that TMS-Cl was injected instead of TMS-Br at 185 °C.

### 2.3. Post-Synthetic Anion Exchange Reaction for Partial Conversion of Cs_2_TiBr_6_ into Cs_2_TiI_6_ NCs

One-quarter of the product obtained from the synthesis of Cs_2_TiBr_6_ NCs was mixed with 10 mL of hexane to prepare Cs_2_TiBr_6_ NC solution (dark red color). Nearly 1.5 mL (a total amount of 6 mL) of TMS-I was injected into the NC solution under Ar flow under vigorous stirring for every 6 h interval, followed by an additional stirring for another 2 days to observe the NC solution color change to black.

### 2.4. Characterization Techniques

The structure, size, and shape determination of the NCs were carried out using the X-ray diffraction (XRD) technique and transmission electron microscope (TEM). High-resolution XRD patterns of the samples drop-casted on glass substrates were recorded on a Malvern Panalytical Empyrean Alpha 1 high-resolution X-Ray diffractometer (Malvern, UK) using Cu K_α_ radiation with λ = 1.5406 Å. Roughly equal concentration of the NCs were used for preparing the drop-casted NC films for the XRD measurements to ensure a valid comparison between the samples. All the patterns were recorded at a slow scan rate (~3° per minute) in order to obtain a high signal-to-noise ratio. The structural information was derived from Rietveld refinement using the General Structure Analysis System (GSAS) software suite [19]. The visualization system for the electronic and structural analysis (VESTA) program was used to draw the crystal structures [20]. The phase purity of the as-synthesized samples was estimated using Rietveld refinement of XRD results considering full refinement of crystallographic and instrumental parameters, as implemented in the GSAS program suite. The low- and high-resolution TEM images were recorded using JEM-F200 (200 kV, JEOL Ltd., Tokyo, Japan). Samples for TEM were prepared by adding a solution of the NCs dissolved in hexane drop-wise on a carbon-coated Cu grid. The solution was allowed to evaporate, leaving behind the NCs. The TEM sampling was carried out in a glovebox to prevent the exposure of the NCs to air before the TEM measurements were performed. Inductively coupled plasma mass spectroscopy (ICP-MS) measurements were conducted with Thermo Scientific iCAP™ RQ equipment (Thermo Fisher Scientific, Waltham, MA, USA). Ionic standard solutions for Ti and Cs were prepared in 2% HNO_3_ using super pure chemicals (Romil-SpA™, Romil Ltd., Cambridge, UK) and ultrapure H_2_O (18.2 MΩ cm, Merck Milli-Q^®^, Merck Millipore, Burlington, MA, USA), and were applied to measure the calibration curves. For the ICP-MS analysis, sample solution containing NCs in hexane matrix was first evaporated to dryness at 40 °C. Then, the solid residue was dissolved in HNO_3_, and finally, diluted with H_2_O to result in a 2% HNO_3_ matrix. Ultraviolet and visible absorption (UV-vis) spectra were recorded with a dual-beam grating Shimadzu UV-1800 absorption spectrometer (Shimadzu Corporation, Kyoto, Japan). PL measurements were performed on an FLS1000 spectrofluorometer (Edinburgh Instruments, Livingston, UK).

### 2.5. Fabrication and Characterization of Cs_2_TiBr_6_ NC-Based Solar Cell in n-i-p Planar Structure

FTO glass substrates (Greatcell Solar, TEC 15, Queanbeyan, Australia), 2 cm × 2 cm, were wet chemically etched with 2M HCl aqueous solution and zinc powder. The etched FTO substrates were then sonicated using an aqueous solution of Hellmanex III solution (2%), acetone, and 2-propanol for 15 min in each step, successively. The substrates were then treated with UV-ozone for 15 min to remove organic residuals and increase hydrophilicity. A 30 nm thick compact TiO_2_ layer (c-TiO_2_) was deposited on the as-prepared patterned substrate by spray pyrolysis of 0.38 M titanium di-isopropoxide bis(acetylacetonate) solution in 2-propanol at 450 °C [21]. We evaluated the thickness of the compact c-TiO_2_ layer by coating a dummy glass with c-TiO_2_ deposited in identical conditions as for the solar cells. Thickness was determined by a surface profiler (Dektak 150 stylus profilometer, measurement error ± 2 nm, Bruker, Billerica, MA, USA). The films were then sintered at 450 °C for 1 h in air. An active layer of Cs_2_TiBr_6_ NCs was then deposited on as-prepared c-TiO_2_ layers by spin-coating a NCs solution (100 mg/mL in hexane) at 1500 rpm for 30 s. The films were directly transferred to a dry nitrogen glovebox. Then, a spiro-OMeTAD layer was spin-coated at 1800 rpm for 30 s. The spiro-OMeTAD solution was prepared by adding 36.2 mg spiro-OMeTAD to 1 mL chlorobenzene and 14.4 µL 4-tBP were stirred using a vortex mixer. Then, 8.7 µL Li-TFSI solution and 14.5 µL FK209 pre-dissolved in acetonitrile were added to the spiro-OMeTAD solution with concentrations of 520 and 300 mg/mL, respectively. Finally, an 80 nm thick gold contact was thermally evaporated on top of the spiro-OMeTAD layer to form the back contact. Evaporation was conducted in a high vacuum (6 × 10^−6^ mbar).

The current density (*J*)*–*voltage (*V*) characteristics were recorded with a Keithley 4250 source-monitor unit (Tektronix, Beaverton, OR, USA), under AM1.5G simulated sunlight (100 mW/cm^2^ irradiance). The illumination was generated through an AAA-solar simulator (Sciencetech Inc., London, ON, Canada) and calibrated using a silicon reference cell.

### 2.6. Third-Harmonic Generation (THG) Microscope Measurements

The sample (Cs_2_TiBr_6_ NCs) was initially prepared on top of a microscopy glass slide and subsequently point-scanned at the focus of a pulsed femtosecond laser (wavelength of 1060 nm, pulse length of 140 fs, repetition rate of 80 MHz). Diffraction-limited focusing was achieved using a high numerical aperture microscope objective (Nikon CFI LU Plan Fluor Epi P, NA of 0.8, infinity-corrected, 50× magnification, Nikon, Tokyo, Japan). The same objective was used to collect the back-scattered nonlinear signal. The scattered light from the fundamental beam was separated using band-pass filters (e.g., Semrock, BSP01-785R-25, and FF01-356/30-25, IDEX Health & Science, LLC, Rochester, NY, USA) and directed onto a cooled photomultiplier tube (PMT). By simultaneously collecting the THG signals as a function of scanning motor positions, a THG map in a region of interest in the sample plane is created. To avoid sample damage during the THG measurements, low power excitation was initially established and then used. For THG verification, appropriate band-pass filters were used (e.g., Semrock, FF01-320/40-25, FF01-385/26-25, IDEX Health & Science, LLC, Rochester, NY, USA).

## 3. Results

**Figure 1:**
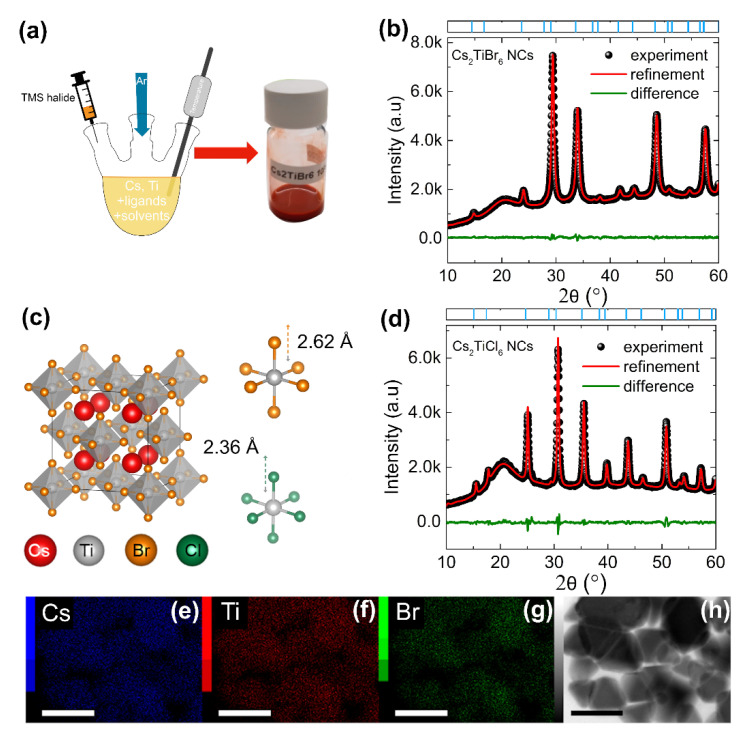
Synthesis and crystal structure. (**a**) Schematic representation of NC synthesis: injection of TMS-Br into precursor solution at high temperature. (**b**) Experimental (black dots), refined (red line), and difference (green line) profiles obtained after the full-pattern Rietveld refinement of the Cs_2_TiBr_6_ NC (synthesized at 185 °C without OlAm) XRD pattern. (**c**) The crystal structure of Cs_2_TiBr_6_, where red, grey, orange, and green spheres represent Cs, Ti, Br, and Cl atoms, respectively. Ti-Br and Ti-Cl octahedra, with the corresponding Ti-halide bond lengths, are also depicted. (**d**) Experimental (black dots), refined (red line), and difference (green line) profiles obtained after the full-pattern Rietveld refinement of the Cs_2_TiCl_6_ NC (synthesized at 185 °C) XRD pattern. (**e**–**g**) EDS elemental (Cs, Ti, and Br) maps of the Cs_2_TiBr_6_ NCs and (**h**) the corresponding STEM image. The scale bar is 100 nm.

To prepare Cs_2_TiBr_6_ NCs, we employed a Schlenk line-based hot-injection route. Briefly, bromotrimethylsilane (TMS-Br) was swiftly injected into an octadecene (ODE) solution containing cesium and titanium precursors along with oleylamine (OlAm) and oleic acid (OA) as the capping ligands, followed by separation and redispersion of the NCs in hexane (see Section 2 for details). By tuning the injection temperature between 90 and 245 °C, the optimal reaction temperature that generated relatively stable NCs was found at 185 °C. The relation between the reaction temperature and NC size, shape, and stability will be discussed in detail below. We selected titanium di-isopropoxide bis(acetylacetonate) as the titanium source. While this is a commonly used precursor for fabricating the TiO_2_ compact layer in perovskite solar cells, its adoption in NC synthesis has not been reported. The injection of TMS-Br resulted in an instantaneous color change of the precursor from nearly transparent to bright red, indicating the formation of the targeted NCs, as shown in Figure 1a. The product could be obtained even without the inclusion of OlAm in the reaction mixture. On the other hand, the direct formation of NCs was not observed when a conventional hot-injection route, with cesium oleate injected into a hot solution of TiBr_4_ in the presence of OlAm and OA, was followed (see Figure S1, Supplementary Materials, SM).

The as-formed Cs_2_TiBr_6_ NCs were first characterized as drop-casted films by the X-ray diffraction pattern (XRD) technique. Figure 1b shows the XRD pattern of Cs_2_TiBr_6_ NCs synthesized at 185 °C. The phase purity and the crystal structural aspects of the NCs were studied by Rietveld refinement of the NC XRD pattern. The refined crystal structure converged with the experimental NC XRD pattern when considering only the Cs_2_TiBr_6_ phase, thus suggesting the phase purity of the Ti-based NCs. These NCs crystallize into a cubic lattice structure with a space group of *Fm*$\bar{3}$*m.* Their crystal structure comprises a solid-state framework of isolated (TiBr_6_)^2−^ octahedra, as shown in Figure 1c. Cs_2_TiBr_6_ adopts K_2_PtCl_6_-type vacancy-ordered A_2_BX_6_ double perovskite structure, where one B^2+^ cation in the double ABX_3_ structure (A_2_B_2_X_6_) is replaced by a vacancy [6,22]. The complete details of the structural parameters obtained from the XRD pattern refinement are presented in Table 1, and Supplementary Tables S1 and S2. The lattice constant value of 10.70 Å for Cs_2_TiBr_6_ NCs closely matches the one derived from Bragg’s law analysis performed on the XRD pattern of bulk Cs_2_TiBr_6_ [8]. Additionally, the Ti-Br bond length (2.62 Å) in this study is consistent with an earlier report [23]. The broadened XRD peaks compared to those of their bulk counterparts imply the presence of smaller crystallites in the as-synthesized Cs_2_TiBr_6_ samples.

Overall, the XRD study reveals that Cs_2_TiBr_6_ NCs adopt the same structure as in their bulk form, and they do not contain any impurity (secondary) phase. We also compared Cs_2_TiBr_6_ NCs with the chloride analogue NCs (Cs_2_TiCl_6_), prepared via a similar synthesis route by injecting TMS-Cl into Cs and Ti precursor solution. As shown in Figure 1d, phase-pure Cs_2_TiCl_6_ NCs crystallize in the same space group as Cs_2_TiBr_6_ (Table 1), with a shorter Ti-Cl bond (2.36 Å) compared to the Ti-Br bond (2.62 Å).

The atomic ratio between Cs and Ti in the NCs shows a negligible deviation from the expected stoichiometric ratio (i.e., Cs:Ti = 2:1), as determined by inductively coupled plasma mass spectrometry (ICP-MS) analysis (see Table S3, Supplementary Materials). The presence of Br and Cl along with Cs and Ti in Cs_2_TiBr_6_ and Cs_2_TiCl_6_ NCs was further confirmed by scanning transmission electron microscopy (STEM)-energy-dispersive X-ray spectroscopy (EDS) mapping (Figure 1e–g, Figure S2 in Supplementary Materials). For example, Figure 1e–g shows that Cs, Ti, and Br elements are uniformly distributed within Cs_2_TiBr_6_ NCs.

**Figure 2:**
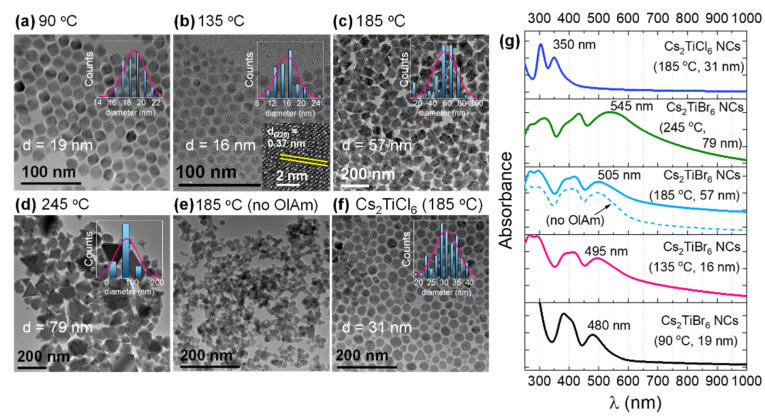
Temperature-dependent morphology evolution and absorption of Cs_2_TiBr_6_ NCs. TEM images of Cs_2_TiBr_6_ NCs synthesized at (**a**) 90 °C, (**b**) 135 °C, (**c**) 185 °C, (**d**) 185 °C, but with no OlAm, and (**e**) 245 °C. (**f**) TEM image of Cs_2_TiCl_6_ NCs synthesized at 245 °C. The corresponding size distributions and the average size (**d**) of the NCs are depicted in the respective panels. The inset of (**b**) represents a HRTEM image of a Cs_2_TiBr_6_ NC, where the spacing between (220) lattice planes is shown. (**g**) The absorption spectra of the NCs present in (**a**–**f**) panels in hexane. The corresponding synthesis temperature, size, and the absorption peak maxima of the NCs are denoted near the absorption spectra.

Figure 2a–e shows the representative transmission electron microscope (TEM) images of Cs_2_TiBr_6_ NCs synthesized at different temperatures. Lower temperatures (90 and 135°) produced spherical or hexagonal-shaped NCs (Figure 2a,b). A relatively high temperature of 185 °C led to the formation of NCs with various shapes (Figure 2c), and large-size prism-shaped particles were obtained predominantly when the synthesis was performed at an even higher temperature (245 °C), as shown in Figure 2d. The inset of Figure 2b shows a high-resolution TEM (HRTEM) image of Cs_2_TiBr_6_ NCs, which depicts the lattice fringes with a separation of 3.7 Å (0.37 nm), corresponding to the (220) lattice planes of Cs_2_TiBr_6_. Additionally, the average size of the NCs gradually increases from 16 to 57 nm, up to 79 nm as the temperature rises, as shown in Figure 2a–e. The summary of this morphological study is presented as Table S4, Supplementary Materials. Our findings are in line with the well-established control of both size and shape by varying synthesis temperature in conventional Pb-based halide perovskite NCs [24,25]. Furthermore, when omitting OlAm during the synthesis of Cs_2_TiBr_6_ NCs at 185 °C, aggregated particles without any definite shape were produced (Figure 2e). This demonstrates the known role of OlAm in controlling the shape of Cs_2_TiBr_6_ NCs. A complex formation between OlAm and Ti^4+^ ions in the case of synthesis with OlAm might alter the reaction rate of Cs^+^ and Br^−^ ions with Ti^4+^ ions and subsequently causes the change in the NC shape. A similar aggregated particle formation was observed for Zr-based double perovskite (Cs_2_ZrBr_6_) NCs when their synthesis was performed without OlAm and only in the presence of OA [26]. Furthermore, Cs_2_TiCl_6_ NCs synthesized at 185 °C in both OA and OlAm have an average size of 31 nm (Figure 2f), which is smaller compared to that of Cs_2_TiBr_6_ NCs synthesized at the same temperature. Since the remaining reaction parameters, such as the Cs:Ti ratio and the concentration of capping ligands, are the same, we speculate that the difference in reactivity of the halide precursor (TMS-X) might have caused the difference in the particle sizes in the two cases. A higher synthesis temperature might be desired for obtaining bigger Cs_2_TiCl_6_ NCs.

Figure 2g depicts the absorption spectra of Cs_2_TiBr_6_ NCs in various sizes obtained at different temperatures. As the NC size increases from 16 to 79 nm, the absorption onsets vary from ~600 to ~650 nm (bandgap of the NCs formed at 185 °C is ~1.9 eV), which is consistent with the red color appearance of the NCs. The bandgap of bulk Cs_2_TiBr_6_ is around 1.8 eV (680 nm) [7]. The exciton peak gradually redshifts from 480 to 545 nm as the NC size increases (Figure S3a, Supplementary Materials), while the bandgap varies from 2.05 to 1.85 eV (Figure S3b, Supplementary Materials). The broadening of this peak in case of 79 nm particles may suggest the reduced quantum confinement as particle size becomes larger, which is consistent with the shift in bandgap towards the value of the bulk perovskite with the increase of the particle size. When the halide changes from Br to Cl, a blue shift in the bandgap is observed for Cs_2_TiCl_6_ NCs (bandgap = 3.4 eV) compared to Cs_2_TiBr_6_ NCs (Figure 2g). The highest VBs of the band structure of Cs_2_TiX_6_ are contributed by halide *n*p orbitals, while Ti 3d orbitals majorly form its lowest CBs [6]. Therefore, the bandgap of Cs_2_TiX_6_ decreases as moving down the halogen group (bandgap: Cs_2_TiCl_6_ (Cl-3p) > Cs_2_TiBr_6_ (Br-4p) > Cs_2_TiI_6_ (I-5p)). Chen et al. reported a bright and sharp PL centered at 700 nm for Cs_2_TiBr_6_-based thin film as a result of its quasi-direct bandgap nature [7]. However, we recorded the PL spectra of Cs_2_TiBr_6_ NCs and did not observe any notable PL signal. While the absence of PL signal from Cs_2_TiBr_6_ NCs contradicts the bright PL observed by Chen et al. [7], it is in good agreement with the feeble and broad PL recently observed from powder samples of Cs_2_TiBr_6_ [8,9]. The very low PL of this material can be attributed to the indirect band gap and parity forbidden transitions associated with the material [8,9].

According to Ju et al., the iodide-containing titanium perovskite Cs_2_TiI_6_ has a band gap of ~1 eV (black body color), suitable for single-junction solar cells [6]. We attempted to synthesize the NCs of this material using our synthesis protocol; unfortunately, we did not succeed in the formation of Cs_2_TiI_6_ NCs. An alternative way to obtain this material is via post-anion (Br^−^ to I^−^) exchange reaction on Cs_2_TiBr_6_ NCs [27]. As shown in Figure S4, Supplementary Materials, the prolonged treatment of Cs_2_TiBr_6_ NCs with a large excess of TMS-I precursor at room temperature resulted in the appearance of a new absorption band with an onset at around 1050 nm (1.18 eV), in addition to the original Cs_2_TiBr_6_ absorption features. Consequently, the NC solution color changes from dark red to black at the end of the reaction (Figure S4, Supplementary Materials), indicative of a partial conversion of Cs_2_TiBr_6_ into Cs_2_TiI_6_ NCs. However, complete conversion into Cs_2_TiI_6_ did not occur even with the further addition of TMS-I into the NC solution. This indicates the limitation of obtaining phase pure Cs_2_TiI_6_ NCs by this approach. Future efforts may thus aim towards new synthetic routes or anion exchange reaction with other iodide precursors to realize phase pure Cs_2_TiI_6_ NCs.

**Figure 3:**
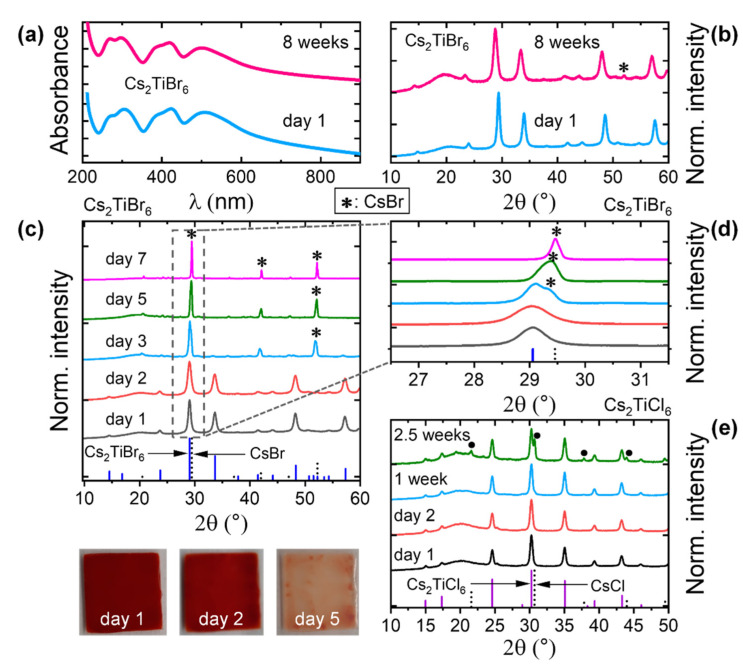
Environmental stability of the NCs. (**a**) Absorption spectra and (**b**) XRD patterns of as-casted Cs_2_TiBr_6_ NC (synthesized at 185 °C without OlAm) films from a hexane suspension, which was kept in ambient conditions up to 8 weeks (~40% RH and ~25 °C). The data were collected at the beginning (day 1) and at the end of the storage time (8 weeks). The photographs are of the Cs_2_TiBr_6_ NC film on day 1, day 2, and day 5 of its storage in ambient conditions. (**c**) The evolution of XRD pattern of Cs_2_TiBr_6_ NC film as a function of time in ambient conditions. The reference XRD patterns of Cs_2_TiBr_6_ (Rietveld refinement data) and CsBr (ICSD code: 01-073-0391) are shown in the same graph. (**d**) A portion of the panel (**c**) is highlighted. (**e**) The evolution of XRD pattern of Cs_2_TiCl_6_ NC film as a function of time in ambient conditions. The reference XRD patterns of Cs_2_TiCl_6_ (Rietveld refinement data) and CsCl (ICSD code: 01-073-0390) are shown in the same graph. The symbols * and **•** represent the reflections corresponding to CsBr and CsCl, respectively. The color change on day 5 indicates the partial loss of TiBr_4_ from the NC film.

There exists dissensus about the ambient stability of Cs_2_TiBr_6_, as contradictory observations on its stability are reported in the limited literature available for this material [7,8,9]. Specifically, while in solution-processed powder samples the degradation peaks appeared in the XRD pattern within a few minutes to hours after exposure to the ambient atmosphere (high instability) [8,9], thin film samples fabricated at high temperature in vacuum remained undegraded under conditions harsher than the ambient environment, i.e., 80% RH for 6 h [7]. Herein, we assess the stability of Cs_2_TiBr_6_ NCs in both solution and film forms. Figure 3a,b show the effect of air storage on both absorbance and crystal structure of Cs_2_TiBr_6_ NC hexane suspension. The suspension remained stable for several weeks with nearly unchanged absorption spectrum, as well as XRD pattern, and only a tiny amount of CsBr impurity (related to the decomposition of Cs_2_TiBr_6_ into CsBr) was observed over 8 weeks. Nevertheless, the degradation was accelerated in film form, as shown in Figure 3c,d. It is evident from Figure 3c that the degradation (CsBr) peaks started appearing in the XRD pattern of Cs_2_TiBr_6_ NC film from day 3 onwards, and the film eventually converted to CsBr after a week of air exposure. Figure 3d shows the (222) lattice plane (at ~29°) region of the XRD pattern of the NCs. After day 2, the CsBr phase was detectable in the XRD pattern and gradually became the majority phase afterward. We have also compared the stability trend for Cs_2_TiBr_6_ NCs synthesized under the same conditions, except the reaction temperature (Figure S5, Supplementary Materials). The small-sized (16 and 19 nm) NC films obtained from 90 and 135 °C syntheses completely transformed into CsBr within 1 day, while the NC film obtained from 185 °C synthesis underwent only negligible degradation within the same timeframe. At the first glance, it seemed that the relatively higher stability of the NC film (185 °C) stems mainly from the bigger (57 nm) size of the particles (higher crystallinity). Nevertheless, the film of 79 nm size NCs (i.e., 245 °C) followed the same stability trend as the ones of 16 and 19 nm particles (90 and 135 °C). The disappearance of a characteristic peak of the Cs_2_TiBr_6_ phase at ~34° on day 2 clearly indicates the degradation of the 79 nm-sized (i.e., 245 °C) NC film (Figure S5, Supplementary Materials). Additionally, except for the 185 °C case, the XRD patterns of the rest of the samples displayed CsBr impurity peaks even in the freshly prepared films, though the majority phase was still Cs_2_TiBr_6_. Therefore, relatively stable and phase pure Cs_2_TiBr_6_ NCs were obtained when synthesized at 185 °C with or without OlAm.

To evaluate the optoelectronic property of as-synthesized Cs_2_TiBr_6_ NCs, we fabricated *n-i-p* planar perovskite solar cell structures by employing the most stable Cs_2_TiBr_6_ NCs formed at 185 °C as the light absorber. The device schematic is FTO/c-TiO_2_/Cs_2_TiBr_6_/spiro-OMeTAD/Au by following previous reported structure for lead-free perovskite solar cells (see the detailed fabrication procedure in Section 2) [28]. Supplementary Figure S6 shows the current density (*J*)*–*voltage (*V*) curves of the champion Cs_2_TiBr_6_ NC-based cell, recorded under 1 Sun condition (100 mW cm^−2^ AM 1.5 G illumination) under forward and reverse bias. The photovoltaic parameters of the best device are summarized and shown in the inset of Supplementary Figure S6. Interestingly, a negligible hysteresis effect of *J–V* curves is noted, which is beneficial for a practical application. However, an extremely low *J*_sc_ (~26 µA cm^−2^) is observed, resulting in an overall low PCE, though *V*_oc_ and FF values are comparable to those of other lead-free double perovskite solar cells [28,29]. This suggests that either the photo-induced charge carrier generation efficiency is extremely low, or the charge carrier recombination rate is extremely fast for this type of Cs_2_TiBr_6_ NCs, leading to the generation of a low photocurrent. Further investigation and engineering work should be conducted to improve the charge transfer dynamics of Cs_2_TiBr_6_ NCs.

The Cs_2_TiCl_6_ NC film exhibited better ambient stability than the bromide counterpart (Cs_2_TiBr_6_): no signs of degradation were observed up to a week of air storage, and the appearance of CsCl (degradation) peaks was observed in the XRD pattern only after two weeks, as shown in Figure 3e. Even then, Cs_2_TiCl_6_ continued to be the majority phase of the NC film. Therefore, Cs_2_TiCl_6_ NCs are clearly more stable than Cs_2_TiBr_6_ NCs under ambient atmosphere. The stability trend of these two NCs is consistent with what is reported for their bulk analogues [8]. The decomposition of Cs_2_TiBr_6_ and Cs_2_TiCl_6_ NCs into CsBr and CsCl respectively, suggests that the degradation pathway in these materials is the loss (evaporation) of the highly volatile TiX_4_ (X = Br and Cl) from Cs_2_TiX_6_, leaving behind CsX in the presence of atmospheric oxygen and water (the NC films are instead stable in an inert environment).

**Figure 4:**
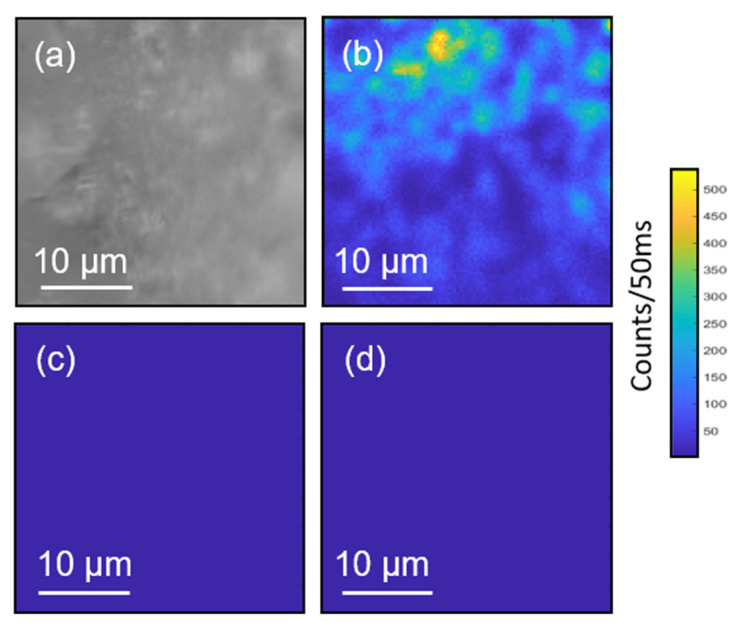
(**a**) Brightfield microscopy image of the drop-casted Cs_2_TiBr_6_ NCs. (**b**–**d**) Scanning THG maps at band-pass filters FF01 356/30-25, 320/40-25, and 385/26-25, respectively. The image resolution is 100 × 100 pixels, and the pixel dwell time is 50 ms.

The centrosymmetric space group of our Cs_2_TiBr_6_ NCs inspired us to investigate possible third-order NLO effects, such as THG [30]. Using a custom-built scanning THG microscope [31], the Cs_2_TiBr_6_ NC films showed strong evidence of THG activity (Figure 4). Here, the NC film was initially prepared on top of a microscopy glass slide via drop-casting method and subsequently point-scanned at the focus of a pulsed femtosecond laser (wavelength of 1060 nm, pulse length of 140 fs, repetition rate of 80 MHz). Figure 4a shows a microscopic region of interest in the perovskite sample. When the same region was scanned at the focal plane, distinct NLO signals were observed (Figure 4b) at the expected THG wavelength (~353 nm). To validate the NLO signals, we re-scanned the same region of interest and collected the NLO signals using different band-pass filters with central wavelengths that are about 30 nm away from the expected THG wavelength. As shown in Figure 4c,d, the NLO signals are very low at the other selected wavelengths. This suggests that the sample is indeed THG-active and could be a promising candidate material in nonlinear photonics. Importantly, to the best of our knowledge, this is the first time that THG is observed for a lead-free perovskite material [12,13,32]. Furthermore, the already reported works on halide perovskites used highly energetic femtosecond pulses (kHz repetition rate) to induce THG activity from their samples. In this work, the THG signals are detectable and potentially useful as a unique imaging contrast. Nevertheless, the THG emissions from our samples are relatively weak since our femtosecond pulse operates in the MHz range. In addition, we found that the THG signals exhibit strong variations. We attribute these variations to inhomogeneities at the sample plane, such as material thickness and orientations of interfaces.

It is worth emphasizing that, while THG spectra and power-dependence curves are traditionally used to demonstrate the THG activity of a sample, we herein show weak THG emission from the samples addressed in a microscopic configuration in contrast to traditional THG setups that perform bulk measurements and use nearly plane-wave excitation geometries. Indeed, the observation and microscopic visualization of THG is already a significant advance in the context of nonlinear optical perovskites. We provide the nonlinear optical scanning maps of the same region in the Cs_2_TiBr_6_ sample using different optical filters. The absence of the THG signals detected at the other wavelengths near the expected THG window is clear evidence of THG, providing essentially the same information of THG spectra. Our choice was mainly restricted by the nature of the sample. The THG power dependence was not shown primarily because of the modest stability of the samples. However, an even more important reason is that the cubic power dependence is indicative of other third-order effects at the same detection window. Hence, this could further mask the real THG behavior. The advantage of our THG-microscope approach over traditional methods lies in the additional visualization capability and higher spatial localization of our technique. The microscopic approach eliminates any other spurious sources of THG, which are easily excited in any centrosymmetric system.

Finally, the results also indicate the possibility of further developing the THG microscopy technique, including its other imaging modalities such as SHG, to study in detail the optical response of individual perovskite NCs, and to reveal how their hierarchical distribution and molecular composition could be exploited for novel sub-wavelength functionalities. Few aspects of these insights are underway and will be reported in a separate work.

## 4. Conclusions

We pioneered the synthesis of eco-friendly and earth-abundant titanium-based halide double perovskite NCs (Cs_2_TiBr_6_) and studied their structural, optical, stability properties, and their potential in optoelectronic applications. The tuning of the hot-injection temperature enables controlling the morphology and size of the as-synthesized Cs_2_TiBr_6_ NCs. The stability study on Cs_2_TiBr_6_ NCs in solution and films revealed that these NCs are vulnerable to ambient moisture and oxygen, and they degrade in film and suspension form within a few days and 8 weeks, respectively. Therefore, the stability outcome of Cs_2_TiBr_6_ in this work is consistent with the trend observed by Kong et al. [8] and Euvrard et al. [9], while it contradicts the excellent stability reported for this material in a highly humid atmosphere [7]. We believe that the discrepancy in Cs_2_TiBr_6_ stability might arise from the material crystallinity differences that stem from the completely different synthesis protocols. To boost the real-life application of Cs_2_TiBr_6_ NC films, it is urgent to improve their environmental stability. One approach may consist in encapsulating the NCs inside a polymer matrix, to protect them from moisture and oxygen [33,34].

The lack of detectable PL and the very low PCE of Cs_2_TiBr_6_ NC solar cells confirms the already observed unsuitability (for bulk films) of this material for photovoltaics. On the other hand, we demonstrated that Cs_2_TiBr_6_ NCs are THG-active. Further investigations to deepen the understanding on the nonlinear optical properties of this material are underway. While a small library of lead-based perovskite materials is NLO-active, Cs_2_TiBr_6_ is the first reported lead-free halide perovskite showing THG. Our work opens an avenue to explore lead-free halide double perovskites for largely overlooked nonlinear photonics.

## Figures and Tables

**Table 1:** Rietveld refinement parameters of Cs_2_TiBr_6_ and Cs_2_TiCl_6_ NCs obtained from XRD data at room temperature. The numbers in parentheses are the estimated standard deviations of the last significant figure.

Material	Space Group	a = b = c/Å	V/Å^3^	^a^ R_wp_	^b^ χ^2^
Cs_2_TiBr_6_	*Fm* $\bar{3}$ *m*	10.70(12)	1224.23(8)	2.35%	1.2
Cs_2_TiCl_6_	*Fm* $\bar{3}$ *m*	10.25(7)	1076.95(5)	2.53%	1.3

^a^ Residual weighting factor, and ^b^ goodness of fit.

## Data Availability

Examples here for study without exterior data: “The data is available on reasonable request from the corresponding author.”/“The data is not available due to [certain reason, privacy, further study, and so on].”/“The data is included in the main text and/or the Supplementary Materials.”

## Supplementary Materials

The following are available online at https://www.mdpi.com/article/10.3390/nano11061458/s1, Figure S1: Photograph of the cesium oleate-TiBr_4_ reaction flask, Figure S2: EDS elemental maps of Cs_2_TiCl_6_ NCs, Figure S3: Particle size-dependent variation of excitonic absorption maximum of Cs_2_TiBr_6_ NCs, Figure S4: The absorption spectrum and the reaction solution color change before and after the anion exchange reaction of Cs_2_TiBr_6_ NCs, Figure S5: XRD patterns of films of Cs_2_TiBr_6_ NCs synthesized at different temperatures, Figure S6: *J–V* curves of the best Cs_2_TiBr_6_ NC-based solar cell. Table S1 and Table S2: Rietveld refinement parameters of Cs_2_TiBr_6_ NCs, Table S3: ICP-MS analysis on Cs_2_TiBr_6_ and Cs_2_TiCl_6_ NCs, Table S4: Summary of temperature-dependent size and shape evolution (TEM analysis) of Cs_2_TiBr_6_ and Cs_2_TiCl_6_ NCs, and a list of abbreviations used in this manuscript.
