# Supplementary material for: Lead-Free Cesium Titanium Bromide Double Perovskite Nanocrystals
Source: Nanomaterials (Basel). 2021 May 31;11(6):1458. doi: 10.3390/nano11061458 (PMC8228098; doi:10.3390/nano11061458)
Supplement: Supplementary file 1 [file nanomaterials-11-01458-s001.zip › nanomaterials-1239984-supplementary.pdf]

Supplementary Material (SM)

Lead-Free Cesium Titanium Bromide Double Perovskite Nanocrystals

G. Krishnamurthy Grandhi [1,†], Anastasia Matuhina [1,†], Maning Liu [1], Shambhavee Annurakshita [2], Harri Ali-Löytty [3], Godofredo Bautista [2] and Paola Vivo [1,*]

1 Hybrid Solar Cells, Faculty of Engineering and Natural Sciences, Tampere University, P.O. Box 541, FI-33014 Tampere, Finland; murthy.grandhi@tuni.fi (G.K.G.); anastasiia.matiukhina@tuni.fi (A.M.); maning.liu@tuni.fi (M.L.)

2 Photonics Laboratory, Physics Unit, Faculty of Engineering and Natural Sciences, Tampere University, FI-33014 Tampere, Finland; shambhavee.annurakshita@tuni.fi (S.A.); godofredo.bautista@tuni.fi (G.B.)

3 Surface Science Group, Photonics Laboratory, Tampere University, P.O. Box 692, FI-33014 Tampere, Finland; harri.ali-loytty@tuni.fi

* Correspondence: paola.vivo@tuni.fi; Tel.: +358-44-3407081

† G. Krishnamurthy Grandhi and Anastasia Matuhina contributed equally to this work.

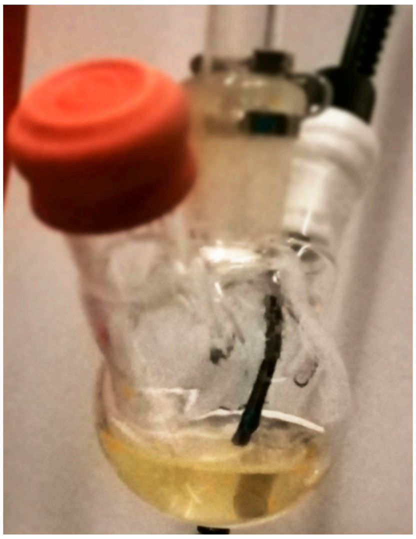

Figure S1. Photograph of the reaction flask (under room light) after hot-injection of cesium oleate into a solution of $TiBr_4$ at 185 ºC. No Cs_2TiBr_6 NC formation took place in this case.

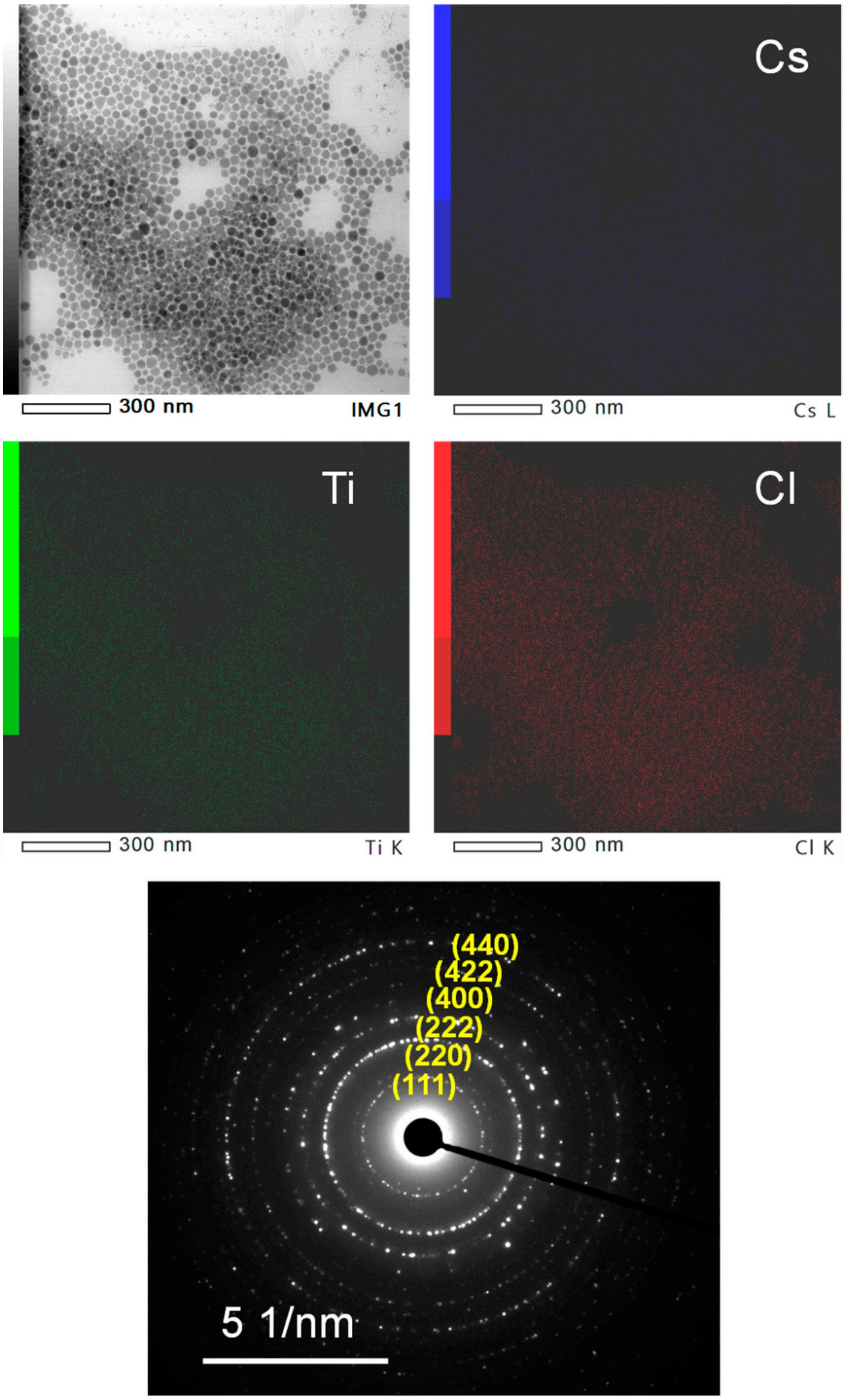

Figure S2. Elemental (Cs, Ti, and Cl) mapping obtained by energy-dispersive X-ray spectroscopy shows the uniformity of the elemental distribution in the Cs_2TiCl_6 NCs and the corresponding scanning transmission electron microscope image. The lattice plane indexing on selective area electron diffraction pattern of the Cs_2TiCl_6 NCs.

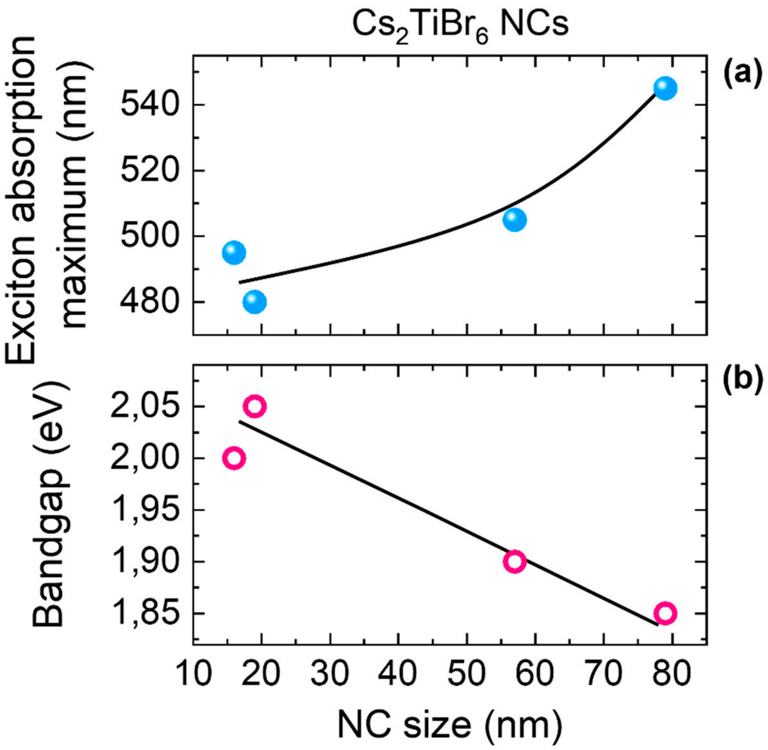

Figure S3. The variation of (a) excitonic absorption maximum and (b) bandgap of Cs_2TiBr_6 NCs as a function of the particle size. The solid lines are just a guide to eye.

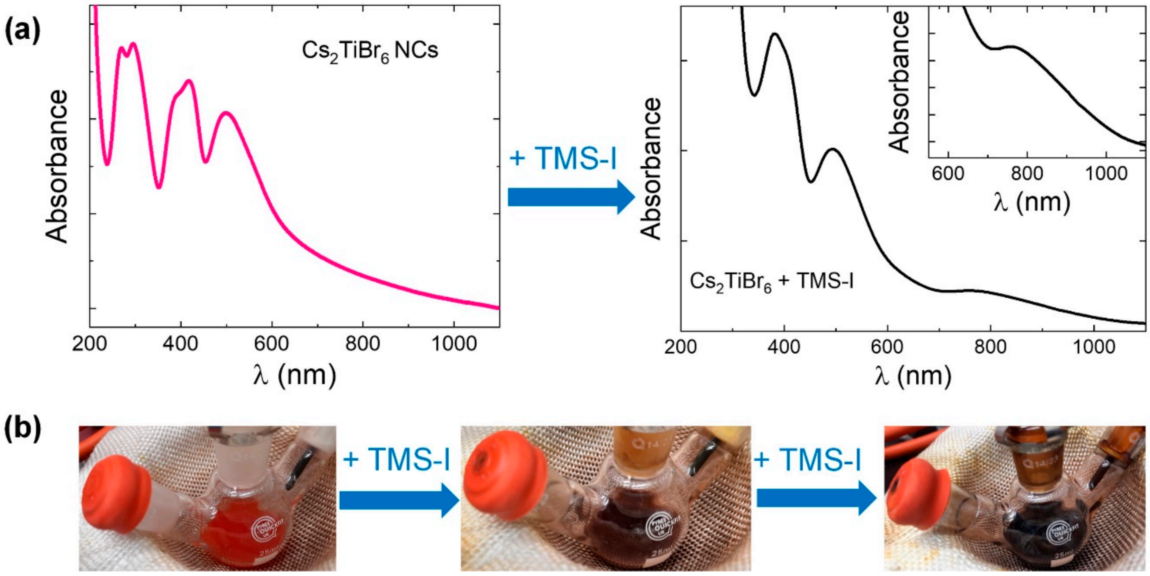

Figure S4. (a) The absorption spectrum before and after the anion exchange reaction of Cs_2TiBr_6 NCs with TMS-I at room temperature. (b) The change in the NC solution color (under room light) as the reaction progress.

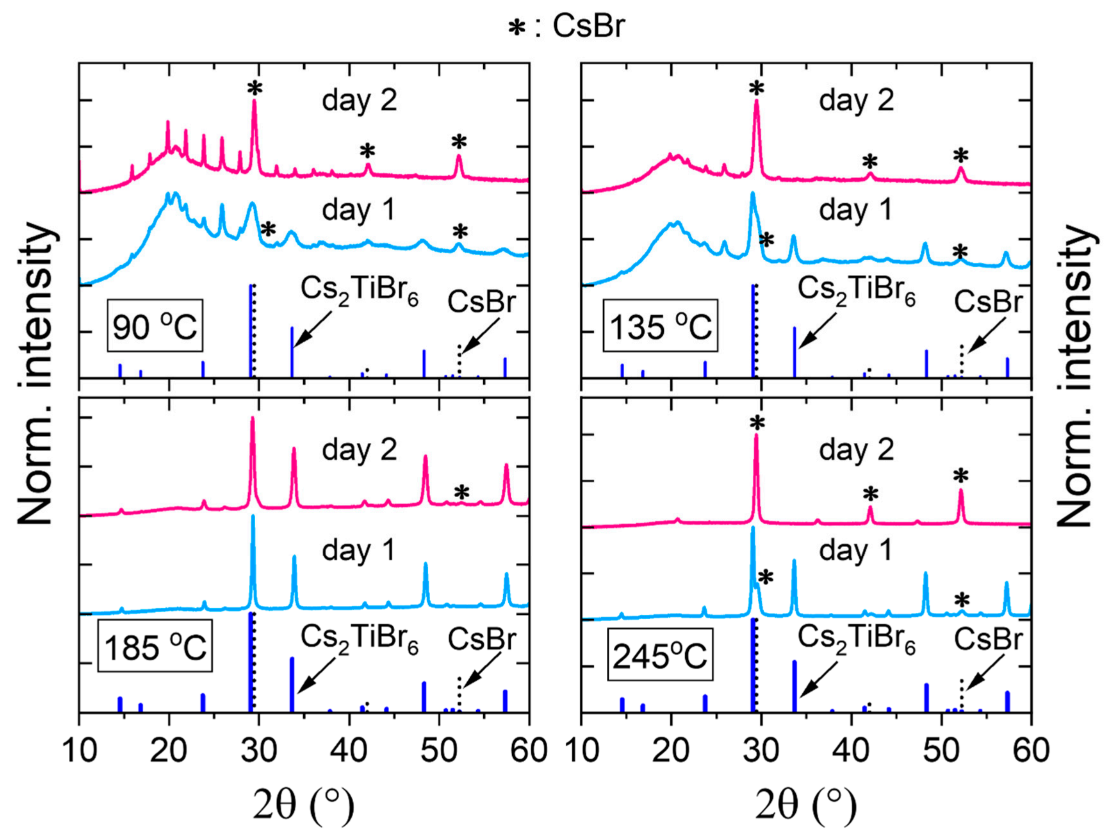

Figure S5. XRD patterns of films of Cs_2TiBr_6 NCs synthesized at different temperatures (given in the respective panels) on day 1 and day 2 of exposure to ambient atmosphere (RH 40%, T=25 °C).

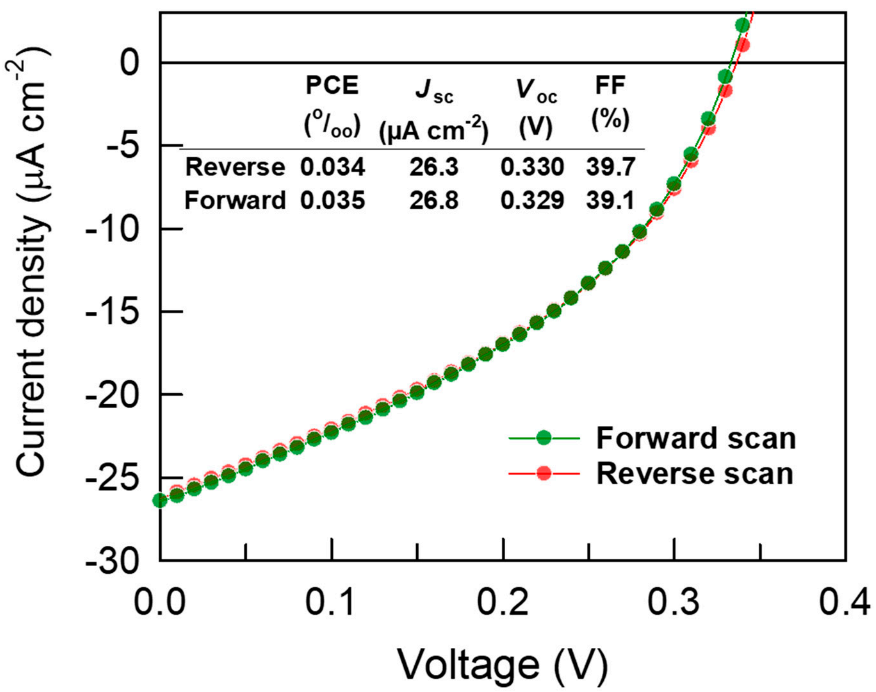

Figure S6. *J-V* curves of the best Cs_2TiBr_6 NC-based solar cell under forward and reverse scans. The scan rate is 20 mV s^{-1} and the active area is 20 mm^2.

Table S1. Structural parameters obtained for Cs_2TiBr_6 phase on performing Rietveld refinement on Cs_2TiBr_6 NC film XRD pattern at room temperature. The numbers in parentheses are the estimated standard deviations of the last significant figure.

Atom	Wyckoff position	*x*	*y*	*z*	$100xU_{iso}(Å)^2$
Cs	8*c*	0.25	0.25	0.25	0.0616(7)
Ti	4*a*	0	0	0	0.0513(4)
Br	24*e*	0	0	0.2451(7)	0.0535(12)

Table S2. Structural parameters obtained for Cs_2TiCl_6 phase on performing Rietveld refinement on Cs_2TiCl_6 NC film XRD pattern at room temperature. The numbers in parentheses are the estimated standard deviations of the last significant figure.

Atom	Wyckoff position	*x*	*y*	*z*	100xU_{iso}(Å)^2
Cs	8*c*	0.25	0.25	0.25	0.0126(17)
Ti	4*a*	0	0	0	0.0126(12)
Cl	24*e*	0	0	0.2451(7)	0.2322(9)

Table S3. Molar ratio determination between Cs and Ti in Cs_2TiCl_6 and Cs_2TiBr_6 NCs by ICP-MS.

Material	Cs (μmoles/l)	Ti (μmoles/l)	Cs:Ti
Cs_2TiCl_6	7.82	3.90	2.00:1.00
Cs_2TiBr_6	8.99	4.96	1.81:1.00

Table S4. Summary of temperature-dependent size and shape evolution of Cs_2TiBr_6 and Cs_2TiCl_6 NCs, from the TEM images presented in Figure 2.

Material	Temperature (ºC)	Avg. Diameter (nm)	Shape
Cs_2TiBr_6	90	19±1.5	Sphere/ellipsoid
	135	16±2.8	Spherical
	185	57±16	Cube
	185 (*no OlAm)*	--	Agglomerated particles
	245	79±44	Prisms
Cs_2TiCl_6	185	31±4.7	Spheres

<u>List of abbreviations</u>

Diisopropoxide bis(acetylacetonate) (TDBA)

Energy-dispersive X-ray spectroscopy (EDS)

Inductively coupled plasma mass spectroscopy (ICP-MS)

Nanocrystals (NCs)

Octadecene (ODE)

Oleic acid (OA)

Oleylamine (OlAm)

Photoluminescence (PL)

Power conversion efficiency (PCE)

Scanning transmission electron microscopy (STEM)

Third-harmonic generation (THG)

Transmission electron microscope (TEM)

X-ray diffraction (XRD)

Bromotrimethylsilane (TMS-Br)

Chlorotrimethylsilane (TMS-Cl)

Iodotrimethylsilane (TMS-I)
